# Modified Total en Bloc Spondylectomy with Self‐Made Intervertebral Hook Blade in Spinal Tumors: A Retrospective Study

**DOI:** 10.1111/os.13748

**Published:** 2023-05-08

**Authors:** Wangzhe Yang, Kun Zhang, Jia Lv, Junjun Bai, Jian Li, Qiaoqiao Tian, Yushan Wang, Zhi Lv, Yi Feng

**Affiliations:** ^1^ Department of the Second Clinical College Shanxi Medical University Taiyuan China; ^2^ Department of Orthopaedics the Second Hospital of Shanxi Medical University Taiyuan China

**Keywords:** Intervertebral hook blade, Modified surgical technique, Spinal tumors, Total en bloc spondylectomy

## Abstract

**Objective:**

Total en bloc spondylectomy (TES) is an important surgical treatment for spinal tumors that can achieve en bloc resection of the affected vertebral body by using the T‐saw. However, the conventional TES process and the surgical instruments currently in use have some inconveniences, which may lead to longer operative times and a higher incidence of complications. To address these obstacles, we developed a modified TES technique using a homemade intervertebral hook blade. The objectives of this study were to describe our modified total en bloc spondylectomy (TES) using a homemade intervertebral hook blade and to assess its clinical effects in patients with spinal tumors.

**Methods:**

Twenty‐three consecutive patients with spinal tumors were included from September 2018 to November 2021. Eleven patients underwent a modified TES using an intervertebral hook blade, and 12 patients underwent a conventional TES using a wire saw. Details of the modified technique for TES were depicted, and the intraoperative blood loss, operative time, and improvement in pain symptom and neurological function measured by visual analog score (VAS) and American Spinal Injury Association (ASIA) score of all patients was reviewed and analyzed. Nonparametric analysis of covariates (ANCOVA) was performed to compare the clinical outcomes between patients treated with modified TES and conventional TES.

**Results:**

The modified TES significantly reduced operative time (F = 7.935, *p* = 0.010) and achieved favorable improvement of neurological function (F = 0.570, *p* = 0.459) and relief of pain (F = 3.196, *p* = 0.088) compared with the conventional TES group. The mean intraoperative blood loss in the modified TES group (2381.82 ml) was lower than that in the conventional TES group (3558.33 ml), although the difference was not statistically significant (F = 0.677, *p* = 0.420).

**Conclusions:**

Modified TES using the intervertebral hook blade can effectively reduce the operation time and intraoperative bleeding, and meanwhile ensure the improvement of neurological function and relief of pain symptoms, suggesting that this modified technique is feasible, safe, and effective for spinal tumors.

## Graphical abstract

A modified total en block spondylectomy using our self‐made intervertebral hook blade for spinal tumors was introduced in this study. This modified technique effectively reduced the operative time and ensured the improvement of neurological function and relief of pain symptoms.

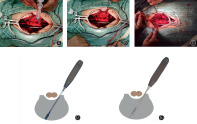

## Introduction

Worldwide, there were approximately 19.3 million new cancer cases in 2020.^1^ Approximately 20% of cancer patients will develop spinal metastases. With the increase in cancer cases and the prolongation of cancer survival, the incidence of spinal metastases will inevitably increase.^2^ The spine is the most common site of metastases within the skeletal system.^3^

Surgery is the most important method to relieve spinal cord compression.^4^ Due to the unique anatomical characteristics and important adjacent tissues of the spine, spine tumors are rarely removed as a whole. In 1997, Tomita et al. developed total en bloc spondylectomy (TES) for spinal tumors to achieve complete resection of the vertebra and minimize local recurrence,^5^ which has greatly improved the surgical management of spinal tumors. Converging studies have demonstrated that TES effectively improves neurological function, relieves pain, and reduces local recurrence in patients with spinal tumors.^6^, ^7^, ^8^ For example, Kato and colleagues treated 23 patients with spinal tumors using TES, and found no tumor recurrences in any of the 23 patients.^7^ Similarly, another study reported a tumor local recurrence of 8.7% in patients who underwent TES.^6^ Huang et al. also found that significant improvement in neurological function was achieved in 13/16 patients and local pain in all patients was reduced after TES.^8^ Conventionally, Tomita's TES utilizes a wire saw to dissect the intervertebral space. However, there are still some inconveniences in the process of conventional TES, which may lead to a long operative time and high incidence of complications. The wire saw is difficult to manipulate and prone to slide to the involved vertebra due to the convex shape of the disc, causing a high risk of tumor cell pollution to the surrounding structures.^8^ Moreover, the wire saw's elastic force may injure the spinal cord during cutting of the spinal column.

In the current study, we innovatively developed a modified TES using our homemade intervertebral hook blade to dissect the intervertebral space and performed a detailed comparison of the clinical outcomes between modified TES and conventional TES. Our aims were: (1) to describe the intraoperative procedure of the modified TES; (2) to evaluate the feasibility and efficacy of our modified TES technique; and (3) to compare the operative time, intraoperative blood loss, and therapeutic effect between the traditional TES and modified TES.

## Methods

### Patients

We included consecutive patients with spinal tumors who were admitted to the Department of Orthopedics of the Second Hospital of Shanxi Medical University from September 2018 to November 2021. All surgical procedures were performed with informed consent from the patients. This study was approved by the local ethics committee of the Second Hospital of Shanxi Medical University [(2023)YX No.(006)].

Twenty‐three patients with thoracolumbar neoplasms who were treated by TES were included. Eleven patients underwent a modified TES using an intervertebral hook blade, and 12 patients underwent a conventional TES using a wire saw. The inclusion criteria were as follows: (1) diagnosed with a spinal tumor with a Tomita type ranging from 4 to 6; (2) single primary vertebral malignancy or benign invasive tumor; (3) in good condition and able to tolerate surgical treatment; and (4) complete clinical data. The exclusion criteria were as follows: (1) metastatic spinal tumor accompanied by systemic metastasis of important organs.

### Patient Evaluation and Data Collection

The baseline clinical information of all patients was reviewed, including age, sex, symptoms, neurological findings, computed tomography (CT) and magnetic resonance imaging (MRI), pathological diagnosis, and tumor location. The number of involved segments was calculated. The Tokuhashi score was used to assess the prognosis of metastatic spinal tumors. Tomita's score was used to determine the therapeutic choice. The visual analog scale (VAS) score and Frankel grade were determined pre‐ and postoperatively to assess the degree of pain symptoms and neurological function. The improvement in VAS score and Frankel grade was assessed by calculating VAS score and Frankel grade difference to quantify the relief of pain and the improvement of neurological function (improvement in VAS = preoperative VAS ‐ postoperative VAS; improvement in Frankel = postoperative Frankel ‐ preoperative Frankel). The operative time and intraoperative blood loss were collected according to the anesthesia note.

The mean age of patients was 51.04 ± 11.94 years and 11 patients were male. The pathological type of spinal tumors was primary in eight patients, metastatic in 14 patients, and unidentified in one patient. The most common was lung cancer (four cases, three in conventional TES and one in modified TES), followed by kidney cancer (three cases, two in conventional TES and one in modified TES), giant cell tumor (two cases, one in conventional TES and one in modified TES), plasma cell myeloma (two cases, one in conventional TES and one in modified TES), chondrosarcoma (two cases in conventional TES), breast cancer (two cases in modified TES). Based on the Tomita system, eight tumors were type 4, 10 tumors were type 5, and five tumors were type 6. There were 18 thoracic tumors, four lumbar tumors and one spanning the thoracolumbar junction. Nineteen patients underwent single‐level TES, one patient underwent two‐level TES, and three patients underwent three‐level TES. Additionally, all patients were followed up.

### Surgical Procedure and Modified Techniques

**FIGURE 1:**
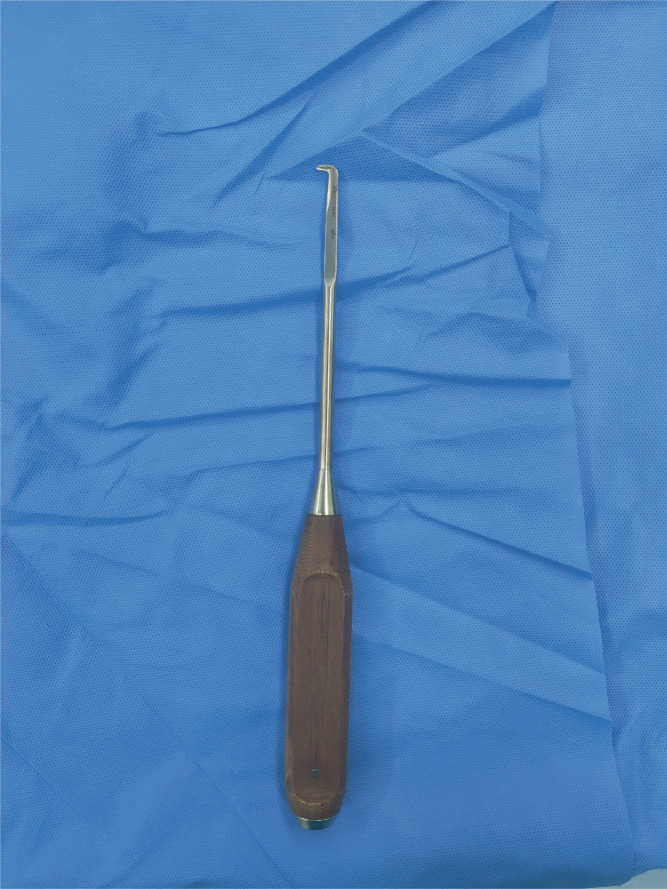
Photograph of our homemade intervertebral hook blade.

Before surgery, routine examination was performed. VAS and Frankel grade were evaluated. None of the patients underwent preoperative segmental arterial embolization. The homemade intervertebral hook blade used in the modified TES operation is shown in Figure 1.

After general anesthesia, the patient was placed in a prone position. An intraoperative radio graph was used to localize the pathological level. A dorsal vertical midline incision was made. The paraspinal muscles were dissected from the spinous processes and retracted to expose the involved vertebra as well as the lamina and zygapophyseal joint of the 2–3 levels superior and inferior to the involved vertebra. If the tumor involved the thoracic vertebra, the same segment of the ribs needed to be exposed. The lateral nerve roots should be ligated and separated. The intercostal nerves and blood vessels were bluntly separated, and the rib heads were removed to expose the pedicle. Pedicle screws were implanted at a minimum of 2–3 levels superior and inferior to the involved vertebra. Intraoperative radiographs were then used to ensure good positioning and direction of the pedicle screws. Then, the whole posterior elements of the spine (the spinous process, the superior and inferior articular processes, the transverse process, and the pedicle) were removed in one piece using the wire saw. The cut surface was sealed with bone wax, and the operative field was rinsed with distilled water to reduce bleeding and minimize contamination by tumor cells. Pleura and soft tissue around the vertebra were separated bluntly from posterior to anterior using fingers until the fingers joined in front of the vertebra, and then it was extended to the discs above and below the involved vertebra. If the tumor involved the lumbar vertebra, the nerve roots at the removal level(s) were retained and carefully dissected from the tumor vertebra to the conjunction with the neighboring nerve roots.

**FIGURE 2:**
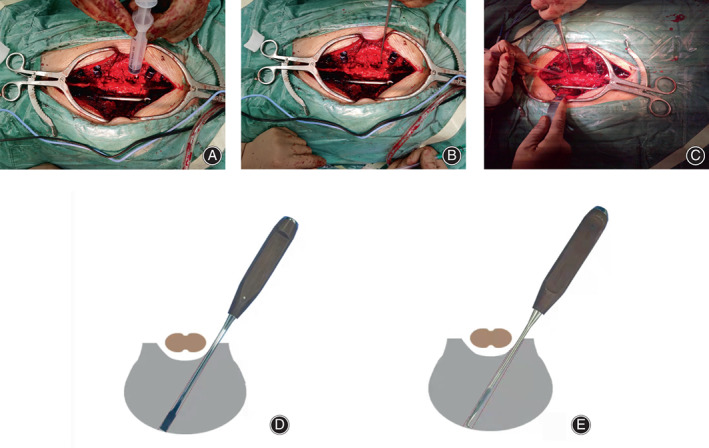
The detailed procedure of the modified TES surgery. (A) Disc position is determined by the syringe needle. (B, D) The osteotome was subsequently placed at the posterior edge of the disc as close as possible to the spinal cord and then inserted obliquely into the disc with an outward tilt angle of 30°. (C, E) The hook blade was inserted into the intervertebral space through the gap created by the osteotome.

In the modified TES group with an intervertebral hook blade, the S‐shaped baffle was subsequently placed in front of the vertebra to protect the prevertebral soft tissues and blood vessels. After the upper disc was identified (Figure 2A), the osteotome was subsequently placed at the posterior edge of the disc as close as possible to the spinal cord and then inserted obliquely into the disc with an outward tilt angle of 30° until it broke through the anterior annulus fibrosus and contacted the baffle (Figure 2B,D). Then, the osteotome was pulled out, and the hook blade was inserted into the intervertebral space through the gap created by the osteotome (Figure 2C,E). After touching the baffle, the hook blade was pulled to the surgeon's side to separate the intervertebral space and the anterior longitudinal ligament. The same operation was performed to cut the lower intervertebral space of the surgeon's side. A titanium rod was temporarily placed to ensure spinal stability. Then, the contralateral upper and lower intervertebral spaces of the involved vertebra were separated using the same operation. After the superior and inferior intervertebral spaces were separated, the surgeon dissected the residual intervertebral soft tissues in the triangle area in front of the spinal cord using a scalpel by softly pulling the dura bilaterally. Using all these operations, the upper and lower intervertebral space of the involved vertebra was completely dissected. In the conventional TES group, Tomita's method was used.^5^

The involved vertebra was gently removed. The operative field was soaked in distilled water for 5 minutes. Then, the surgeon selected a titanium mesh with an appropriate length and filled it with allogeneic bone graft. The titanium mesh and posterior instrumentation were then installed. Intraoperative radiography was finally used to ensure the appropriate implantation of titanium mesh and posterior instrumentation. The operative field was rinsed with distilled water again. Finally, a drain was placed, and the wound was closed by layers. The involved vertebra was sent to the pathology department for further pathological examination after the operation.

Postoperative spinal radiographs were reviewed to observe the internal fixation. Antibiotics should be administered prophylactically for 3 days. VAS and Frankel grade were assessed on the third day after surgery.

### Statistical Analysis

Group differences in demographics were compared with two‐sample t tests for continuous variables and chi‐square tests for categorical variables. Due to the non‐normal distribution of data, nonparametric analysis of covariates (ANCOVA)^9^ was used to analyze the group difference in operative time, intraoperative blood loss, and improvement in VAS score and Frankel grade between the modified and conventional TES groups. All of the statistical analyses were performed using SPSS v25.00 (IBM).

## Results

### Patient Data

**FIGURE 3:**
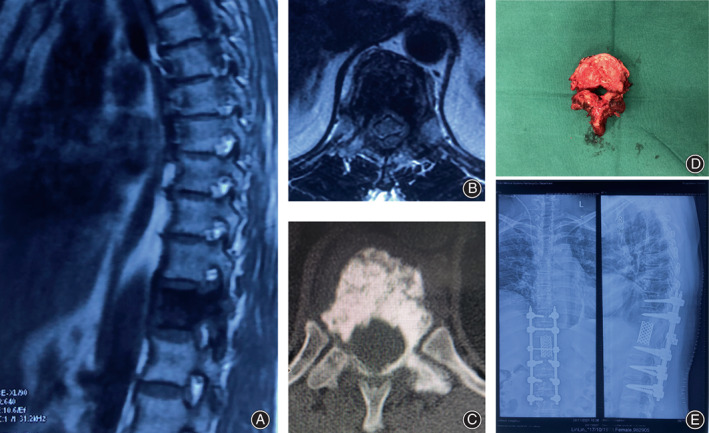
Illustrative case 22. (A) Sagittal MRI shows that the T11 vertebra is involved in an osteogenic tumor in a 48‐year‐old female patient. (B, C) Axial CT and MRI scans show the pedicle involved under epidural and paraspinal extension of the mass. (D) A specimen of breast cancer metastasis at T11. (E) Postoperative radiograph reveals dorsoventral spinal instrumentation.

All patients underwent TES successfully, as illustrated in Figure 3. The demographic and clinical information of 23 patients with spinal tumors were summarized in Table S1. Among the 23 patients, eight had primary spinal tumors, and 14 had metastatic spinal tumors. Nineteen patients had a single vertebra resected, one had two vertebrae and three had three vertebrae. The neurological function of all patients was improved or maintained after surgery. Thirteen patients showed grade 1–2 improvement. Seven patients developed complications, including five cases of pleural rupture, one case of cerebrospinal fluid leakage, and one case of instrumentation failure. Eleven patients underwent a modified TES, and 12 underwent a conventional TES. There was no significant difference in age, sex, number of involved segments, preoperative VAS score, or preoperative Frankel grade between the modified TES group and the conventional TES group (Table 1).

**TABLE 1:** Demographic differences between groups at baseline

					Preoperative Frankel (n, %)			
Groups	Age (years; Mean, SD)	Sex (Male/female)	Number of involved segments (Mean, SD)	Preoperative VAS (Mean, SD)	B	C	D	E
Modified TES (n = 11)	48.91 (8.91)	6/3	1.27 (0.65)	5.82 (2.18)	0	4 (36.4)	2 (18.2)	5 (45.5)
Conventional TES (n = 12)	53.00 (14.29)	5/7	1.33 (0.78)	6.25 (0.62)	2 (16.7)	2 (16.7)	5 (41.7)	3 (25.0)
t/chi^2^	0.815	0.583	−0.202	0.658	4.417			
*p*	0.424	0.445	0.842	0.518	0.220			

### Follow‐Up

The average follow‐up time was 20.22 ± 10.62 months (range: 2–48 months). No clinical evidence of local recurrence was found in all patients during the follow‐up. Eight patients died during the follow‐up (four in conventional TES group and four in modified TES group). Specifically, case 1 died of respiratory failure because of pulmonary metastasis at 24 months. Case 4 died from postoperative complications of rectal cancer operative at 18 months. Case 12 died of cerebral hemorrhage 12 months after surgery. The death of other patients was due to systemic disease progression.

### Surgical Outcomes

The nonparametric ANCOVA test revealed a significant decrease in operative time in the modified TES group compared with the conventional TES group (F = 5.649, *p* = 0.027). This difference remained significant after correcting for the effects of the number of involved segments and the type of primary tumor (F = 7.935, *p* = 0.010). However, there was no significant difference in intraoperative blood loss and the improvement in VAS score and Frankel grade between the modified and conventional TES groups (Table 2). To verify the results, we conducted all analyses again using a general linear model (a parametric method) and found that the result in operative time remained significant, suggesting that the result is reliable (Table 2).

**TABLE 2:** Differences in operative time, blood loss, and VAS improvement between the modified TES and conventional TES groups using nonparametric ANCOVA and a general linear model

Indexes		Modified TES (n = 11)	Conventional TES (n = 12)	Nonparametric ANCOVA		GLM	
				F	*p*	F	*p*
Operative time (Min)	Model 1	245.00 (40.25)	307.08 (66.45)	5.649	0.027*	7.172	0.014*
	Model 2	245.00 (40.25)	307.08 (66.45)	7.911	0.010**	11.512	0.005**
	Model 3	245.00 (40.25)	307.08 (66.45)	7.935	0.010**	8.978	0.007**
Blood loss (ml)	Model 1	2381.82 (905.34)	3558.33 (2426.67)	0.738	0.400	2.284	0.146
	Model 2	2381.82 (905.34)	3558.33 (2426.67)	0.750	0.396	2.147	0.158
	Model 3	2381.82 (905.34)	3558.33 (2426.67)	0.677	0.420	1.870	0.187
VAS improvement	Model 1	3.82 (1.67)	4.67 (0.49)	3.235	0.086	2.863	0.105
	Model 2	3.82 (1.67)	4.67 (0.49)	3.239	0.086	2.726	0.114
	Model 3	3.82 (1.67)	4.67 (0.49)	3.196	0.088	2.258	0.149
Frankel improvement	Model 1	0.45 (0.52)	0.67 (0.65)	0.574	0.457	0.733	0.401
	Model 2	0.45 (0.52)	0.67 (0.65)	0.644	0.431	0.696	0.414
	Model 3	0.45 (0.52)	0.67 (0.65)	0.570	0.459	0.448	0.512

*Note*. Nonparametric ANCOVA and general linear models were used to evaluate the differences in operative time, blood loss, and VAS improvement between the modified TES and conventional TES groups, separately. The mean and standard deviation are shown for two groups, as well as *p* values to indicate statistically significant group differences. Model 1 did not include any covariates. Model 2 included the number of involved segments as covariates. Model 3 included the number of involved segments and the type of primary tumor as covariates.

Abbreviations: GLM, general linear model; VAS, visual analog scale; TES, total en bloc spondylectomy.

* *p* < 0.05.

** *p* < 0.01.

### Complications

In the modified TES group, two patients developed a pleural burst during the surgery which was repaired immediately. One patient required reoperation due to instrumentation failure at 24 months after surgery. In the conventional TES group, three patients developed a pleural burst which was repaired immediately. One patient experienced a cerebrospinal fluid leak, and the patient was asked to lie in a Trendelenburg position.

## Discussion

The emergence of TES has changed the concept of surgical treatment of spinal tumors, effectively reducing the local recurrence rate of spinal tumors and improving neurological function, but it also has some shortcomings, such as a long operation time and a large amount of bleeding. In this study, we modified the TES with a homemade intervertebral hook blade. We found that the modified TES significantly reduced the operative time and improved neurological function and pain relief, suggesting that the modified TES is a feasible, safe, and effective technique for treating spinal tumors.

### Intraoperative defects of traditional TES

TES surgery proposed by Tomita originally used a wire saw to dissect the pedicle and upper and lower discs of the involved vertebra through a “one‐step” approach from anterior to posterior. However, some inconveniences in the process have limited the utility of TES in clinical practice. First, it is difficult to maintain the wire saw in a fixed position, and it is prone to slide to the infected vertebra due to the convex shape of the disc, which may lead to a high risk of tumor cell contamination. On the other hand, the wire saw is difficult to manipulate. When cutting the spinal column by an “anterior to posterior” approach, the wire saw cannot stop just before completion, and its elastic force may injure the spinal cord as soon as cutting is completed.^10^ Some teams, including the author's, have modified the surgical instruments, such as disc puncture and osteotome.^8^, ^10^, ^11^, ^12^ For example, Huang's team performed TES by using T‐saw and self‐made osteotome together.^8^ Another study used T‐saw and the disc puncture needle with a sleeve to dissect the discs.^10^ These modified techniques facilitate the process of the TES to some degree, but they still have some shortcomings because T‐saw is still needed. Some surgeons dissected discs using osteotome. But the disc needs to be osteotomized multiple times, which may lead to incomplete separation of the intervertebral tissues, failure to remove the vertebra, and accidental injury to the spinal cord.^12^ Particularly, for tumors in the lower lumbar region, TES using wire saws or osteotomes can be challenging because the lumbar vertebra is situated at a deep location and is larger than other vertebrae. Our homemade hook blade can dissect the anterior longitudinal ligament and intervertebral space tissue with only one cut, prevent damage to the adjacent vertebral endplate and spinal cord, and is also more convenient to apply to the lower lumbar spine.

### Advantages of modified TES in operation time

The modified TES significantly reduced the operative time. In our study, the average operation time of modified TES group was 245.00 ± 40.25 min, which was significantly longer than that of the conventional TES (307.08 ± 66.45 min). Moreover, the operation time in our study was also shorter than that of Ji and colleagues reported at 424.3 ± 108.1 min in 23 patients using the conventional TES technique.^13^ Two previous studies developing modified TES techniques reported the operation time of 365 min^14^ and 432 min^8^ separately. Previous studies have suggested long operative time and excessive intraoperative blood loss as independent risk factors for postoperative infection.^15^, ^16^ Thus, the reduction in operative time in the modified TES group may contribute to reducing the risk of postoperative infection. Additionally, the decrease in operative time is inevitably accompanied by a decrease in blood loss. Although the between‐group difference was not statistically significant, the mean blood loss in the modified TES group (2488.89 ml) was lower than that in the conventional TES group (3533.33 ml) in our study. However, compared with the previous literature, patients in our study had relatively more blood loss.^8^, ^13^, ^14^ The mean blood loss reported in previous studies ranged from 2300 to 2514 ml.^8^, ^14^ In another study that included 23 participants, 21 cases received preoperative trans‐arterial embolization and the mean intraoperative blood loss was 1883.3 ml. The blood loss of the other two cases was 2800 and 4000 ml, respectively.^13^ Thus, the reason for the relatively higher blood loss in our study may be that patients did not receive preoperative trans‐arterial embolization. This may also be one of the reasons why there was no significant difference in blood loss in this study.

### The characteristics of modified TES in improving neurological function

The neurological function of all patients was improved or maintained after surgery in our study. Of the 23 patients, 13 achieved grade 1 or 2 improvement. This finding agrees with previous reports,^17^, ^18^ in which the neurological function of 20 patients treated with TES was improved or maintained, and nine of 11 patients who could not walk reached functional independence after surgery.^17^ Moreover, in line with a previous study,^17^ the pain symptoms of all patients were significantly relieved in our study. The improvement in VAS score or Frankel grade was equivalent between the two groups, suggesting that the modified TES achieved the same effect in pain relief and functional improvement as conventional TES. In the modified TES group, two patients developed a pleural burst during the surgery which was repaired immediately. One patient required reoperation due to instrumentation failure at 24 months after surgery. In the conventional TES group, three patients developed a pleural burst. One patient experienced a cerebrospinal fluid leak. The patient with cerebrospinal fluid leak was asked to lie in the Trendelenburg position. The incidence of complications is consistent with previous reports.^6^, ^19^ For example, in a study that included 23 subjects, 15 patients (65.2%) developed at least one perioperative complication, with the most common being wound infection and ileus.^6^ Another study observed 29 cases of postoperative cerebrospinal fluid leakage (21%), and 11 cases of surgical site infection (8%) among 140 patients.^19^ Furthermore, no clinical evidence of local recurrence was found in all patients during the follow‐up. Therefore, the recurrence rate was not analyzed in our study. Additionally, it is generally considered that patients with Tokuhashi scores below 9 have poor outcomes in TES treatment. However, some researchers suggest that this criterion is not applicable for patients with spinal metastasis of lung cancer.^20^, ^21^ Consistent with this, in our study, we found that a patient with lung cancer spinal metastasis (No. 12) with a preoperative Tokuhashi score of 6 received significant improvement in pain symptoms, neurological function and quality of life after TES treatment. Furthermore, to correct the effect of this patient, we conducted the analyses repeatedly after removing the data of patient No. 12 and found that the results remained significant.

### Limitations and Strengths

There are several limitations in our study. First, this is a retrospective study with a small sample size. Further prospective large‐scale studies with follow‐up are needed to validate our results. Second, sometimes, some surgeons dissect the affected vertebra from the vertebral body rather than the intervertebral space.^22^ However, our homemade hook blade is not suitable for cutting bone. Therefore, it is not applicable for this type of surgery. Furthermore, it is necessary to invent surgical instruments that can be used in a variety of surgical situations.

Despite the recognized limitations, this study has some advantages. First, this study introduced a modified TES technique using a homemade intervertebral hook blade that can dissect the intervertebral space and conveniently protect the spinal cord. Furthermore, this is a case–control study with comprehensive patient data, which suggests the clinical advantages of modified TES over traditional TES thoroughly.

### Conclusion

In conclusion, we found that our modified TES with a homemade intervertebral hook blade can effectively reduce the operative time, decrease intraoperative blood loss to a certain extent, and ensure the improvement of neurological function and relief of pain symptoms. TES with the modified technique is technically feasible, safe, and effective for spinal tumors.

#### Authorship Declaration

All authors listed meet the authorship criteria according to the latest guidelines of the International Committee of Medical Journal Editors. All authors agree with the manuscript.

## Authors' Contributions

Wangzhe Yang and Yi Feng designed the manuscript. Wangzhe Yang was responsible for searching literature, statistical analysis, and drafting the manuscript. Wangzhe Yang, Kun Zhang, Jia Lv, Junjun Bai, Jian Li, Qiaoqiao Tian, Yushan Wang, Zhi Lv, and Yi Feng made contributions to critical revisions for important intellectual content. Wangzhe Yang and Yi Feng revised and finalized the manuscript. All authors read and approved the final manuscript.

## Conflict of Interest Statement

The authors declare that the research was conducted in the absence of any commercial or financial relationships that could be construed as a potential conflict of interest. The authors have no conflict of interest to report.

## Supporting information


**Table S1.** Patient data.
