# Supplementary material for: Modified Total en Bloc Spondylectomy with Self‐Made Intervertebral Hook Blade in Spinal Tumors: A Retrospective Study
Source: Orthop Surg. 2023 May 8;15(6):1599–606. doi: 10.1111/os.13748 (PMC10235171; doi:10.1111/os.13748)
Supplement: Supplementary file 1 — Table S1. Patient data. [file OS-15-1599-s001.docx]

Table S1 Patient data

|  |  |  |  |  |  |  |  | VAS | | Frankel | |  |  |  |  |  |  |
| --- | --- | --- | --- | --- | --- | --- | --- | --- | --- | --- | --- | --- | --- | --- | --- | --- | --- |
| No. | Group | Age | Sex | Diagnosis | Location | OT, min | BL, ml | pre | post | pre | post | Tomita classification | Tomita score | Revised Tokuhashi score | Complication | Follow-up time | Death |
| 1 | TES | 76 | M | Fibrosarcoma | T3-T5 | 355 | 2500 | 6 | 1 | B | C | 5 | / | / | None | 24 | Yes |
| 2 | TES | 59 | F | Lung cancer | T11 | 320 | 1000 | 7 | 3 | D | E | 5 | 5 | 9 | None | 48 | No |
| 3 | TES | 54 | M | Kidney cancer | L2 | 325 | 8000 | 6 | 1 | D | E | 4 | 3 | 13 | None | 39 | No |
| 4 | TES | 48 | F | Rectal cancer | T10 | 305 | 4500 | 7 | 2 | E | E | 5 | 5 | 12 | Pleural burst | 18 | Yes |
| 5 | TES | 50 | M | Plasma cell myeloma | T11 | 380 | 5500 | 6 | 1 | D | E | 5 | / | / | None | 33 | No |
| 6 | TES | 56 | F | Lung cancer | T7 | 255 | 1300 | 6 | 1 | C | D | 4 | 5 | 10 | Pleural burst | 25 | No |
| 7 | TES | 26 | F | Kidney cancer | T10 | 195 | 1800 | 6 | 1 | D | E | 5 | 3 | 13 | None | 25 | No |
| 8 | TES | 31 | M | GCT | T11 | 320 | 2000 | 5 | 1 | E | E | 5 | / | / | None | 24 | No |
| 9 | TES | 73 | F | Chondrosarcoma | L1 | 285 | 2200 | 7 | 2 | C | C | 5 | / | / | None | 26 | No |
| 10 | TES | 57 | M | Chondrosarcoma | T3-T5 | 420 | 7600 | 6 | 2 | B | D | 6 | / | / | Pleural burst | 9 | Yes |
| 11 | TES | 55 | F | Thymic carcinoma | L4 | 325 | 4500 | 7 | 2 | D | D | 5 | 5 | 10 | None | 24 | No |
| 12 | TES | 47 | M | Lung cancer | T3 | 260 | 1500 | 6 | 1 | B | D | 6 | 5 | 6 | Cerebrospinal fluid leakage | 7 | Yes |
| 13 | MTES | 47 | F | Cholangiocarcinoma | T12 | 225 | 1500 | 6 | 2 | C | D | 4 | 5 | 11 | None | 13 | Yes |
| 14 | MTES | 57 | M | Urothelial carcinoma | T11-L1 | 335 | 3000 | 8 | 3 | C | D | 6 | 5 | 11 | None | 5 | Yes |
| 15 | MTES | 58 | M | Lung cancer | T12 | 300 | 3000 | 5 | 2 | C | D | 6 | 5 | 9 | None | 12 | Yes |
| 16 | MTES | 39 | M | Osteoblastoma | T12 | 210 | 3000 | 7 | 2 | E | E | 5 | / | / | Instrumentation failure | 24 | No |
| 17 | MTES | 53 | M | Plasma cell myeloma | T7 | 240 | 1700 | 6 | 2 | D | E | 4 | / | / | Pleural burst | 22 | No |
| 18 | MTES | 49 | F | Angiosarcoma | T10 | 215 | 2700 | 5 | 2 | C | D | 4 | 5 | 9 | None | 2 | Yes |
| 19 | MTES | 55 | M | Unidentified | T6 | 210 | 2000 | 10 | 3 | E | E | 4 | / | / | None | 20 | No |
| 20 | MTES | 60 | M | Kidney cancer | T9 | 210 | 4300 | 7 | 2 | E | E | 5 | 3 | 13 | None | 17 | No |
| 21 | MTES | 40 | F | Breast cancer | T9-T10 | 250 | 1200 | 4 | 2 | E | E | 6 | 4 | 12 | Pleural burst | 16 | No |
| 22 | MTES | 48 | F | Breast cancer | T11 | 250 | 1800 | 2 | 1 | E | E | 4 | 2 | 15 | None | 16 | No |
| 23 | MTES | 32 | F | GCT | L2 | 250 | 2000 | 4 | 1 | D | D | 4 | / | / | None | 16 | No |
| Note. TES: total en bloc spondylectomy; MTES: modified total en bloc spondylectomy; OT: operative time; BL: blood loss; GCT: giant cell tumor. | | | | | | | | | | | | | | | |  |  |
